# Animal Mechanism and Physiology

**Published:** 1840-04

**Authors:** 


ART. VII.—*Animal Mechanism and Physiology.* By JOHN H. GRISCOM, M.D., Professor of Chemistry, &c.—*New York*, 1839. 18mo, pp. 357.

THIS is another work having the same laudable object, and by no means deficient in qualifications for success. It is principally compiled from Sir C. Bell's "Animal Mechanics," Dr. Arnott's "Physics," and Dunglison's "Physiology;" and therefore it is not likely to contain much harm. The author's physiology is, however, a little antiquated in many instances; and we cannot recommend it as a guide on that subject. We are surprised that our friends on the other side of the Atlantic pay so little regard to the style of typography, and especially to the manner in which their works are illustrated. From the announcement of "Woodcuts by Butler," in the title-page, we anticipated at least mediocrity in their execution; but we are obliged to declare that a worse set of illustrations never came before us. With one or two exceptions, the cuts are badly drawn, worse engraved, and printed worst of all. Indeed, the whole getting-up of the book is far inferior to that which we are now accustomed to see in the cheap popular works of our own country. In this respect, Messrs. Chambers have done wonders; and it is the neatness of their cheap and unpretending treatise which has led us to speak rather strongly on the subject of Messrs. Harper's more bulky and expensive one. We should certainly recommend our professional brethren in the States to send their drawings to England by the Great Western, and we will warrant their being well transferred to wood. Our only fear is that, when they are brought into use, they will be spoiled in the printing; as it is well known that the best wood-engravings may be, in bad hands. We beg the excuse of our friends in New York for our plain speaking; and assure them that it will give us sincere pleasure to receive a correction in the shape of a well-executed series of woodcuts of *any* description.

